# Pain chronification risk assessment: advanced phenotyping and scoring for prediction and treatments tailored to individualized patient profile

**DOI:** 10.1007/s13167-024-00383-3

**Published:** 2024-11-15

**Authors:** Igor Martuliak, Olga Golubnitschaja, Lubos Chvala, Marko Kapalla, Miroslav Ferencik, Michala Bubeliny, Michal Venglarcik, Ladislav Kocan

**Affiliations:** 1 Department of Algesiology, Slovak Medical University Bratislava, F.D. Roosevelt University General Hospital, Banska Bystrica, Slovakia; 2 Predictive, Preventive and Personalised (3P) Medicine, University Hospital Bonn, Rheinische Friedrich-Wilhelms-Universität Bonn, 53127 Bonn, Germany; 3 Department of Psychiatry, Slovak Medical University Bratislava, F.D. Roosevelt University General Hospital, Banska Bystrica, Slovakia; 4 Department of Anaesthesiology, Slovak Medical University Bratislava, F.D. Roosevelt University General Hospital, Banska Bystrica, Slovakia; 5 The Department of Anesthesiology and Intensive Care, Pain Center, East Slovak Institute of Cardiovascular Disease, Faculty of Medicine, Pavol Jozef Safarik University, Kosice, Slovakia

**Keywords:** Chronic pain, Pain chronification prediction, Algesiology, Predictive preventive personalized medicine (PPPM/3PM), Low-grade inflammation, Phenotyping, Patient stratification, Diabetes, Flammer syndrome, Health-to-disease transition, Risk assessment, Systemic risks, Shifted regulation, Pain perception and sensitivity, Mitochondria, Survey, Scoring, Personalized protection, Primary and secondary care, Health policy, Questionnaire

## Abstract

Acute pain is a physiologic, protective life-important warning neurological signal indicating multi-level tissue modulations caused by a broad spectrum of health adverse events such as stress overload, mechanical trauma, ischemia–reperfusion, sterile and infection-triggered inflammation, single- and multi-organ damage, acute and chronic wounds, tissue remodeling and degeneration, amongst others. On the other hand, pain chronification results in a pathologic transformation from the protective pain signaling into persistent debilitative medical condition with severe consequences including but not restricted to phenotype-specific behavioral patterns, reduced quality of life, and cognitive and mood disorders. Who is predisposed to an increased vs. decreased pain sensitivity and to the pain chronification? The motivation of personalized medicine that “same size does not fit all” is getting obvious also for an advanced approach in algesiology. Consequently, an in-depth patient stratification is essential for the paradigm change in overall pain management from currently applied reactive medical services to the cost-effective predictive, preventive, and personalized medicine (PPPM/3PM) in primary (reversible damage to health and targeted protection against health-to-disease transition) and secondary (personalized protection against disease progression) care. To this end, specifically innovative concepts of phenotyping elaborated in this study play a crucial role in patient stratification for predicting pain-associated outcomes, evidence-based targeted prevention of the pain chronification, and creation of treatment algorithms tailored to individualized patient profiles.

**Supplementary Information:**

The online version contains supplementary material available at 10.1007/s13167-024-00383-3.

## Preamble

Acute pain is a physiologic, protective life-important warning neurological signal indicating multi-level tissue modulations caused by a broad spectrum of health adverse events such as stress overload, mechanical trauma, ischemia–reperfusion, sterile and infection-triggered inflammation, single- and multi-organ damage, acute and chronic wounds, tissue remodeling and degeneration, amongst others. Life-threatening consequences of an impaired protection by pain signaling can be demonstrated on patients with diabetic history and progressing secondary pathologies. Due to neuropathic degenerative processes and associated downregulation of the pain perception as a frequent complication of advanced diabetes mellitus (DM) type 1 and type 2, the affected patient cohort suffers from impaired wound healing, silent myocardial infarction, and sudden cardiac arrest more frequently than the general population [1–4].

In contrast, the pain sensitivity may get significantly increased in otherwise healthy individuals with Flammer syndrome phenotype (FSP) whose wound healing is slowed down [2, 5].

An enhanced pain perception is considered an instrumental adaptive mechanism in FSP individuals for phenotype-specific natural protection, e.g., against avoidable mechanical traumata and as “warning signals” for the health-to-disease transition. To this end, shifted regulation of the senses-associated receptors such as temperature, pain, and thirst perception is linked to highly increased stress sensitivity typical for the FSP carriers suffering from strongly pronounced stress-provoked vasospastic reactions [6–10].

For example, occurring more frequently than in the general population, headache and migraine with aura are characteristics of young individuals with FSP predisposed to lacunar ischemic brain lesions [11] and glaucomatous damage of the optic nerve [6].

On the other hand, pain chronification results in a pathologic transformation from the protective pain signaling into persistent debilitative medical condition with severe consequences including but not restricted to phenotype specific behavioral patterns, reduced quality of life, and cognitive and mood disorders. Who is predisposed to an increased vs. decreased pain sensitivity and to the pain chronification? The motivation of personalized medicine that “same size does not fit all” is getting obvious also for an advanced approach in algesiology. Consequently, an in-depth patient stratification is essential for the paradigm change in overall pain management from currently applied reactive medical services to the cost-effective predictive, preventive, and personalized medicine (PPPM/3PM) in primary (reversible damage to health and targeted protection against health-to-disease transition) and secondary (personalized protection against disease progression) care. To this end, specifically innovative concepts of phenotyping elaborated in this study play a crucial role in patient stratification for predicting pain-associated outcomes, evidence-based targeted prevention of the pain chronification, and creation of treatment algorithms tailored to individualized patient profiles.

## Pain chronification attributes potentially relevant for phenotyping and patient stratification

Chronic pain can be characterized in a reduced way by the term “sensitisation,” which means a kind of “hyperexcitation” of the nervous system, i.e., its excessive sensitivity to stimuli and excessive reactivity. This process is associated with a number of restructuring changes of the nervous system in its neurobiochemical metabolism, function, and structure (so-called “pathological neuroplastic changes”), which can lead to dysfunction, and, in extreme cases, to the failure of transmission and inhibitory nociceptive systems. The reason for the chronification of acute pain with its transition to chronic pain is not fully clear, and we still do not know why chronic pain appears in some people and not in others. In this process, not only the intensity and duration of the current pain play an important role but also the gradual failure of the functional inhibitory mechanisms of pain perception in the central nervous system (CNS). It seems that at least 2 conditions must be present for the emergence of chronic pain, which we can basically divide into peripheral and central. A peripheral cause means the presence of sufficiently long-term and intense nociceptive afferentation of pain impulses from the area of damage to the CNS, which can also have an iatrogenic cause in the case of poor diagnosis or treatment of the cause of pain, or it is a mistake on the part of an uncooperative or treatment-ignoring patient.

A central cause means the presence of a predisposed, “latent” situation of sensitization of CNS structures. After robust clinical experience of more than 30 years of algesiology practice, we assume that sensitization is caused by a chronic, sufficiently intense, and sufficiently long-lasting stressful situation, such as chronic post-traumatic stress disorder, e.g., after surviving serious life situations and unprocessed retained emotions. A common cause is also craniocerebral trauma with the development of chronic post-concussion syndrome. The CNS can thus reach a state of violation of the dynamic balance of its activity, which is conditioned by the disruption of the action of neurotransmitters with the influence of excitatory molecules predominating over inhibitory ones. The result is often only a discrete “hyperexcitation,” which does not yet have to be manifested by significant physical or psychological symptoms in the clinic. In the clinical profile of the patient, we can observe only subtle signs of irritation, both psychological, such as nervousness, irritability, tearfulness, insomnia, anergy, and problems with concentration, and, on the one hand, physical, where with a careful anamnesis and examination, we can register the persistence of neck (or other) muscle shortening, a reduced pain threshold, but also sensory sensations (hypersensitivity to sounds or light). Various manifestations of vegetative imbalance are also present, such as oppression, palpitations, hypertensive disease (often drug-resistant), reflux disease, and GIT dyskinesia—including syndrome irritable colon, sweating, disorders of peripheral circulation, and many other manifestations of the predominance of sympathetic tone. However, these symptoms are sufficiently characteristic of the state of CNS sensitization that we can conclude from their presence the risk of chronic pain.

Several researchers were evaluating the role of psychological conditions that can increase the chance of chronification of the pain. The authors Masselin-Dubois et al. evaluated the role of anxiety, depression, and catastrophizing in chronic postsurgical pain of arthroplasty or breast surgery for cancer [12]. In their study, linear regression models showed that state anxiety and pain magnification as dimensions of catastrophizing predicted chronic pain intensity. McCowat et al. conducted a systematic review about psychological predictors of acute and chronic pain in women following breast cancer surgery [13]. Across twelve studies, they identified anxiety, depression, and distress as the most tested and significant predictors of acute or chronic pain. Similarly, Giusti et al. performed a robust systematic review and meta-analysis of psychological and psychosocial predictors of chronic postsurgical pain [14]. They included 83 studies into narrative synthesis and 41 studies into meta-analysis. The narrative synthesis showed that evidence about the effect of psychological predictors is heterogeneous but with few expected predictors such as optimism, state anxiety, and psychological distress for chronic postsurgical pain. In contrast, their meta-analysis showed that state anxiety, anxiety trait, mental health, depression, catastrophizing, kinesiophobia, and self-efficacy have weak but significant associations with chronic postsurgical pain. So far, there is a strong premise that psychological and psychosocial factors could be significant factors alongside somatic conditions in the chronification of the pain. There is a strong assumption that chronification of the pain can be partly predicted before planned surgical procedures. The aim of this study was to create and verify an instrument Risk of Pain Chronification Questionnaire (RPCQ) with potential to predict chronification of the pain as we have outlined in our previous publication [15].

## Working hypothesis

Sensitization of the CNS is an essential basis for the emergence and development of chronic pain. Published experience from the clinical practice of treating chronic pain indicates that it generates a relatively homogeneous group of symptoms in most patients who suffer from it. At the same time, it is also possible to identify relatively similar etiological, mostly psychological connections. Chronic pain is a source of serious suffering for a significant amount of the adult population and at the same time represents a huge economic burden for the whole society. Also for this reason, it is very effective to try to find a tool for the primary prevention of the chronification of acute pain and its transition into chronic pain. This assumption is based on the principles of predictive, preventive, and personalized medicine (PPPM/3PM), as it allows to detect the risk of developing chronic pain even before its first symptoms appear [16]. Based on that, we created our questionnaire RPCQ with dedicated and well-considered items. These items were expected to be generated by consensus among clinicians and researchers experienced with chronic pain treatment.

## Methods

### Item design

Items (questionnaire questions) for the RCPQ were carefully generated using consensus statements regarding findings from treatment outcome studies of patients with chronic pain or after surgery, literature review of the books, and published articles about chronification of the pain. This process is in line with the recommendations by Cronbach and Meehl that advised researchers to articulate the theoretical concept of an instrument before developing and testing it empirically [17]. We intended to adapt the concept of overinclusiveness, so we did not significantly restrict the number of items to be included in a final version. In total, 27 items were generated, focused on depressiveness, anxiousness, somatic symptoms of anxiety, genital difficulties, pain symptoms, repeated negative medical outcomes, and heartburn. All proposed items were thoroughly discussed with a group of experts possessing multi-professional expertise in acute and chronic pain treatments, psychology, anesthesia, and psychiatry, among others. The proposed version of the questionnaire was tested in cognitive interviews. The instrument was composed of two separate parts: (1) The statements of each item (e.g., “Have you ever experienced any severe pain?”), (2) “If yes, how much was it uncomfortable for you? (0—Not at all, 1—Slightly, 2—Moderately, 3—Very, 4—Extremely)”. Those who did not have experiences listed in the statements left the answer unresponded. We decided to exclude the possibility of answering “no” to make the process of filling out the instrument easier and faster for older patients, who are more frequent in ambulances for pain treatment where we were gathering data. Those who did not experience statements described in the items did not have to answer. Moreover, items 2, 21, and 27 had very specific but similar expressions for the second part of each item (for example: “If yes, how much did your perception of the new pain get worse?”).

### Data collection

The data were collected from September 2022 to October 2023. Respondents were invited to participate in the research during their first visit of the chronic pain out-patient clinic for pain treatment. The patients were informed about the research and had to fill out The Risk of Pain Chronification Questionnaire (RPCQ) and the short form of The Brief Pain Inventory (BPI-SF) while waiting for a medical doctor and the pain treatment. The process of filling out the questionnaires did not significantly prolong the time for receiving a standard treatment. Immediately after the first visit and completing the RPCQ and BPI-SF, respondents were asked to visit an ambulance in half a year for the second time to fill BPI-SF again to see if the treatment had effect. The criteria for participation included (a) age over 18 years and (b) having experienced pain. The patients were not paid for participation.

### Samples

We obtained data from 230 respondents. Out of this number, only 207 respondents finished the whole RPCQ and were suitable for an exploratory factor analysis. At the start of performing the regression analysis, applying student *t* tests and repeated analysis of variance, we excluded 114 respondents from those analyses because they did not finish at least 75% of the items for each individual evaluation factor defined as a subgroup of items specific for corresponding factor. Descriptives of the samples are presented in the results of each analyses.

### Additional instrument

The Brief Pain Inventory (BPI-SF) contains 9 items that are self-administered and used to evaluate the severity of the patient’s pain and the impact of this pain on the patient’s daily functioning. The patient is asked to rate their worst, least, average, and current pain intensity; list current treatments and their perceived effectiveness; and rate the degree that pain interferes with walking ability, work, mood, relations with other persons, enjoyment of life, sleep, and general activity, on a ten-points scale. Inventory was translated and adapted to numerous languages including Chinese, Italian, German, Greek, Norwegian, Japanese, Spanish, and Slovak [18]. According to some authors, BPI-SF can be considered as a reliable instrument. Moreover, some studies already tested construct validity of the BPI-SF by confirmatory factor analysis. In the literature, there were proposed two factor solutions. The first solution operated with three factors named as pain intensity, activity interference, and affective interference; meanwhile, the second solution considered affective interference as an independent factor from the previous two. Atkinson et al. support the conclusion that both solutions are usable for HIV/AIDS and cancer populations [19]. Moreover, Lapane et al. and Tan et al. reported that the second solution has greater validity for patients with non-cancer pain [18, 20]. We decided to use the Slovak version of BPI-SF and its two-factor solution in our study. The first factor is called pain severity. This first factor measures the experiencing of different forms of pain. The second factor is called interference, and its items measure how much pain interferes with daily function of the patients. We decided to call the second factor in our study “a functional ability.”

### Exploratory factor analysis

All analyses were conducted using JASP software version 0.18.1.0. (https://jasp-stats.org/). In the first step, we performed exploratory factor analysis (EFA) with principal axis factoring to determine the validity and factor structure of the instrument. This kind of factor analysis is suitable for assessing theoretical interesting latent constructs rather than to test specific hypotheses. For an EFA, it is appropriate to operate with interval or at least quasi-interval data, which can be assumed for data that we collected. For extracting the number of factors, we implemented oblimin rotation. According to Browne, an oblique rotation permits factors to be correlated, which orthogonal rotation does not, and is thus more representative for data where it is reasonable to assume that different factors in the same instrument in fact correlate to some degree [21]. Also, we performed the Kaiser-Meyer Olkin (KMO) measure of sampling adequacy and the Bartlett’s Test of Sphericity, which indicates if the correlations between items are significantly different from zero, as well as the determinant, checking for a reasonable level of correlation. The scree test was performed to visually inspect the number of factors that precedes the last major drop in eigenvalues. Parallel analysis was implemented in JASP to compare the obtained factor solution with one derived from data that is produced at random with the same number of cases and variables. The final number of factors was decided based on parallel analysis, scree plot, and Kaiser’s rule, as well as the interpretability of the factor solution.

In the second step, we computed the mean subscale scores for the two BPI-SF subscales—severity of the pain (computed items 3, 4, 5, and 6) and functional ability (computed items 10, 11, 12, 13, 14, 15, and 16) and mean subscale scores for four factors of our RPCQ (severe pain and sensitization, visceral somatization, health-related anxiousness, and health-related depressiveness). Into analyses, we included only respondents who answered at least 75% of items for each scale. We estimated the multiple linear regression model and used age and four RPCQ factors as predictors for severity of the pain severity and functional ability of the patients after 6 months of standard treatment. Multiple linear regressions (Enter method) were used to identify the significant predictors. Standardized regression coefficients were obtained using the JASP (soft. version 0.18.1.0). Multicollinearity was controlled by means of tolerance (TOL < 0.10) and variance inflation factor (VIF > 10). On the basis of these criteria, none of the analyzed variables showed multicollinearity.

In the third step, we performed two student *t* tests of two paired samples and several repeated measures of variance. In the first place, we performed two student *t* tests to verify if there was a statistically significant difference between pre-test and post-test after 6 six months of standard care for two subscales of BPI-SF: pain severity and functional ability. After that, we performed several repeated analyses of variance to check if there is a statistically significant difference in scores of pain severity and functional ability between pre-test and post-test (after 6 months of standard treatment) according to higher or lower overall score of RPCQ and its factors (2 × 2 repeated measures). These two samples were defined from the overall score of RPCQ and its factors (22 items proposed by EFA). The first sample consisted of respondents who scored ≥ 25th percentile of overall RPCQ score or its four factors, while the second sample consisted of respondents who scored ≤ 75th percentile of overall RPCQ score or its four factors.

## Results

### Results of exploratory factor analysis

In multivariate statistics, exploratory factor analysis (EFA) is a statistical method used to uncover the underlying structure of a relatively large set of variables. EFA is a technique within factor analysis whose overarching goal is to identify the underlying relationships between measured variables. We used EFA to identify most characteristic factors defined as subgroups of items specific for corresponding factor explaining our proposed items of RCPQ. Patients included in the study were represented by 69 men with a mean age of 57.89 years and 138 women with a mean age of 60.94 years. In the starting phase of analysis, we got a proposal in JASP software to use a “three-factor” solution with deleting items 4, 5, 16, 9, and 11, based on parallel analysis.

**Fig. 1:**
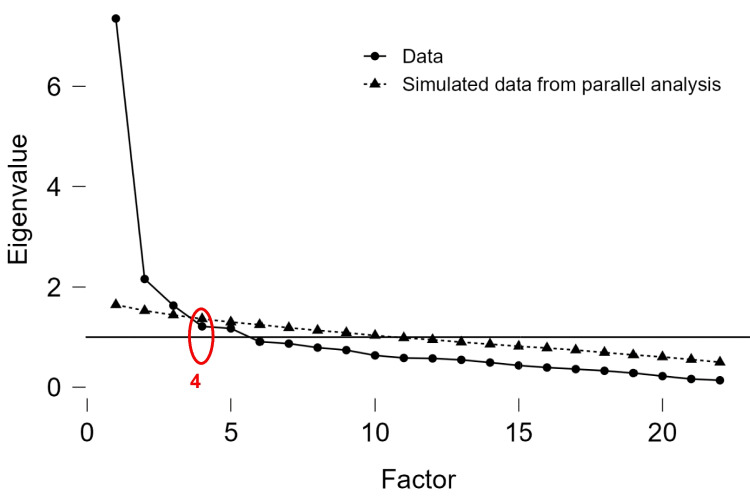
Scree plot of eigenvalue for four-factor solution. It shows that the optimal number of components to retain for further analysis is 4 in this particular case

After several rounds of evaluation, a “four-factor” solution with deleting items (questions) 9, 11, 12, and 15 was settled as the optimal one within the current study (see Fig. 1). In this “four-factor” solution, there were no cross-loadings and all items had loadings only on one factor over 0.4. In this “four-factor” solution, we found that overall Kaiser–Meyer–Olkin value (KMO) was 0.869, and the KMO values for individual items were ranging from 0.688 to 0.940. Bartlett’s test of sphericity (χ2 (2072.656), df = 231.000, *p* < 0.001) indicated that correlations between items were sufficiently large for conducting EFA. Parallel analysis, Kaiser’s criterion, and the scree plot converged on a four-factor solution. These four factors had eigenvalues from 1.215 to 7.354 (Fig. 1).

Supplementary Table 1 shows the loadings of items on the four-factor model. The four factors together explained 47% of the variance. The 1st factor was named as “Severe pain and sensitization,” with seven items such as “Have you ever experienced any severe pain?” or “Have you ever experienced sudden and extremely intense physical pain that significantly worsened your perception of the pain?” (items included in the 1st factor: 1., 2., 3., 4., 5., 10., and 11.). The 2nd factor was related to “Visceral Somatization,” with four items such as: “Have you ever had problems with pain in the genital area?” or “Have you ever had difficulty with bowel movements (diarrhea, constipation)?” (items included in the 2nd factor: 6., 7., 8., and 9.). The 3rd factor was recognized as “Health related anxiousness,” with six items such as: “Have you ever had such anxiety that you couldn't concentrate on your daily activities or work?” or “Have you ever had disturbing thoughts about possible hospitalization that would limit you in your normal activities?” (items included in the 3rd factor: 12., 13., 14., 15., 16., and 17.). The 4th factor was recognized as “Health related depressiveness” with items such as: “Have you ever felt like nothing was bringing you joy anymore to the point that it was limiting your normal functioning?” or “Have you ever been troubled by feelings of guilt that limited you in your normal functioning?” (items included in the 4th factor: 18., 19., 20., 21., and 22.).

### Internal consistency of the RPCQ and its factors

We evaluated the reliability of the overall RPCQ score and its four factors with analysis of the internal consistency. The internal consistencies for overall score of RPCQ and its four factors were satisfying (ranging from 0.701 to 0.897). Correlations between identified factors in EFA are shown in Supplementary Table 2. The internal consistency of the whole scale was α = 0.897.

### The overall score of RPCQ and its factors as predictors for severity of the pain and functional ability of the patients after 6 months of standard pain treatment

We excluded 91 respondents from this analysis, because they responded on less than 75% of the items of each variable. Descriptives and results of the regression analyses are presented in Supplementary Tables 3–5. The model of the four factors of the RPCQ and age predicted severity of the pain after 6 months of standard treatment (*R*^2^ = 0.201, *p* < 0.001). Similarly to the severity of the pain, in the case of functional ability of the patients after 6 months of the standard treatment, the model of the overall mean score of RPCQ and age predicted a slight proportion of the variance (*R*^2^ = 0.111, *p* < 0.001).

### Comparison between pre-test and post-test and repeated measures analysis of variance based on lower or higher score of overall RPCQ or its factors for severity of the pain and functional ability of the patients over 6 months of standard pain treatment

Similarly to regression analysis, we excluded 91 respondents from the analysis of *t* tests and repeated measures analyses of variance. Descriptives and results of these comparative analyses are presented in Supplementary Tables 6–10. We found significant differences for reporting the severity of pain and decrease in functional ability according to the overall score of RPCQ and its factors between pre-test (visit of the ambulance for chronic pain treatment) and post-test (after 6 months of the standard treatment). Patients who visited the ambulance for standard treatment of the pain after 6 months reported a significantly lower severity of the pain and decrease in their functional ability. Also, we found statistically significant differences for reporting of the pain and functional ability between pre-test and post-test after 6 months of standard treatment according to lower or higher RPCQ overall score. Notably, we found that there was a significant difference for reporting of the pain severity and functional ability between pre-test and post-test according to lower or higher health-related depressiveness. Respondents with lower scores in health-related depressiveness (The Fourth factor of RPCQ) reported less pain and less decrease in their functional ability after 6 months of standard treatment of the pain. The differences between pre-test and post-test were not significant for the overall score of RPCQ or its three other factors.

## Data interpretation

### The established survey is considered instrumental for individualized prediction of pain chronification

Overall, the study performed has confirmed our working hypothesis that specifically CNS sensitization caused by persistent stress overload may lead to pain chronification. Mental health plays a critical role including clinical manifestation of the sympathetic over-excitation. The established survey is considered instrumental for individualized prediction of the pain chronification followed by patient stratification and targeted prevention in the cohort of individuals at high risk. Our study clearly demonstrated that specifically psychological factor (mental health, anxiousness, and depressiveness) is crucial for predicting pain chronification in vulnerable subpopulations such as individuals undergoing surgical treatments that is well in consensus with previously published research data [14]. To this end, RPCQ scoring was of great predictive power for stratifying patients at high versus low risk of developing chronic pain: the respondents who scored lower on health-related depressiveness during pre-test were less likely to report severe pain or decrease in functional ability after 6 months of standard treatment (post-test). Contextually, we are optimistic about potential clinical utility of the RPCQ testing, although its follow-up validation is essential using bigger patient cohorts.

### Psychological factor is crucial

The aim of this study was to create and evaluate validity and reliability of an inventory for predicting chronification of the pain. Patients with chronic pain demonstrate a relatively homogeneous group of symptoms with a strong dominance of the psychological factor [15]. Based on that, first, we created a RPCQ with 27 items (not shown). Those were generated by consensus among clinicians and researchers experienced with chronic pain treatment. All proposed items of RPCQ were based on literature about clinical research of predictors of chronification of the pain and clinical experiences of clinicians experienced with daily treatment of such pain. Moreover, we were inspired by Örebro Musculoskeletal Pain Questionnaire (OMPG) created by Linton and Boersma [22]. Their OMPG predicts the risk of long-term disability and sick leave after musculoskeletal injury. Similarly, the Acute Low Back Pain Screening Questionnaire (ALBPSQ, Hurley) and the Vermont Disability Prediction Questionnaire (Hazard) were developed to stratify patients with low, medium, or high risk to developing chronic pain [23, 24]. Those questionnaires have great value but are based primarily on current or past somatic pain items. It has none or very insufficient number of items for psychological or psychosocial variables. However, particularly psychological and psychosocial aspects are crucial according to our experience. Therefore, we decided to add more corresponding items reflecting the health-related anxiety or depression, primarily for the reasons that chronic pain is psychologically associated with symptoms of an anxiety-depressive disorder.

### The four-factor solution proposed is the particular innovation of the current study

The primary version of the RPCQ with 27 items was administered to patients who came for the first time to take a standard treatment of pain into an out-patient clinic for pain treatment. A four-factor solution with adequate fit and acceptable factorial validity and internal consistency in the final version of our developed and here presented RPCQ with 22 items in total (Table 1) and respective scores for low risk, medium risk, and high risk of pain chronification (Table 2). The corresponding four factors are severe pain and sensitization, visceral somatization, health-related anxiousness*,* and health-related depressiveness. The first two factors: severe pain and sensitization and visceral somatization represent mostly the somatic aspects. These two factors characterize the pathophysiological and clinical condition associated with experiencing chronic pain. Severe pain and sensitization are key factors for the process of pain chronification. Sensitization of the nervous system is the pathophysiological basis of chronic pain. The cause of its occurrence is most often unprocessed psychological trauma in the past, usually already in childhood, states of long-term suffering in life, and concussion or other damage to the central nervous system [15]. As a result, a combination of pathological neurochemical changes of the CNS is characterized mainly by disruption of neuronal functions and varying degrees of excessive sensory excitation of the brain. In this way, a new pathological state of activity, especially of the sensory part of the CNS, with excessive reaction even to small subthreshold stimuli develops. However, this situation is, of course, transferred to the function of the whole body, which is in a similar state to the situation of hyperexcitation of the brain. This process is called somatization, which is the second essential factor in our questionnaire. It is the transfer of information about the state of mind and CNS to the whole body. Somatization is realized via the neuronal pathway of the limbic system, hypothalamus, and pituitary gland, while the neurohypophysis maintains excessive sympathetic tone in the organism during CNS sensitization, and the adenohypophysis, in turn, maintains the hormonal situation of the archetypal combative state “fight or flight” [15, 25]. Thanks to the process of somatic and especially visceral somatization, the patient’s body generates a relatively varied spectrum of clinical symptoms. According to their presence, we can diagnose the presence and degree of sensitization of the nervous system and the risk of chronic pain. In principle, the basic pathophysiological difference between acute and chronic pain is that, although the source of pain is present in both types of pain, in acute pain, there is no clinically significant sensitization of the CNS, and thus pain inhibitory mechanisms work normally [26].

**Table 1:**
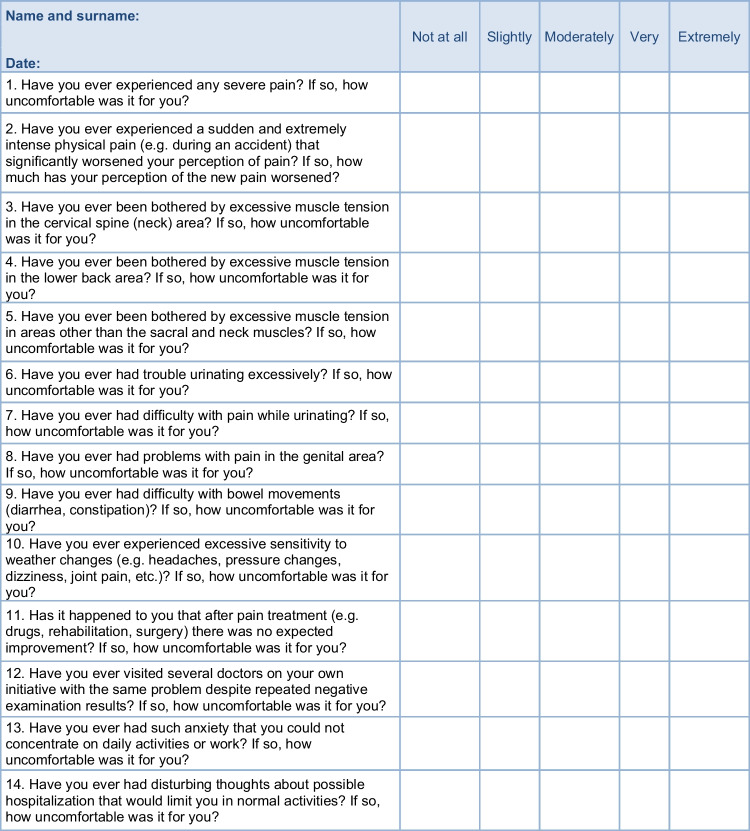

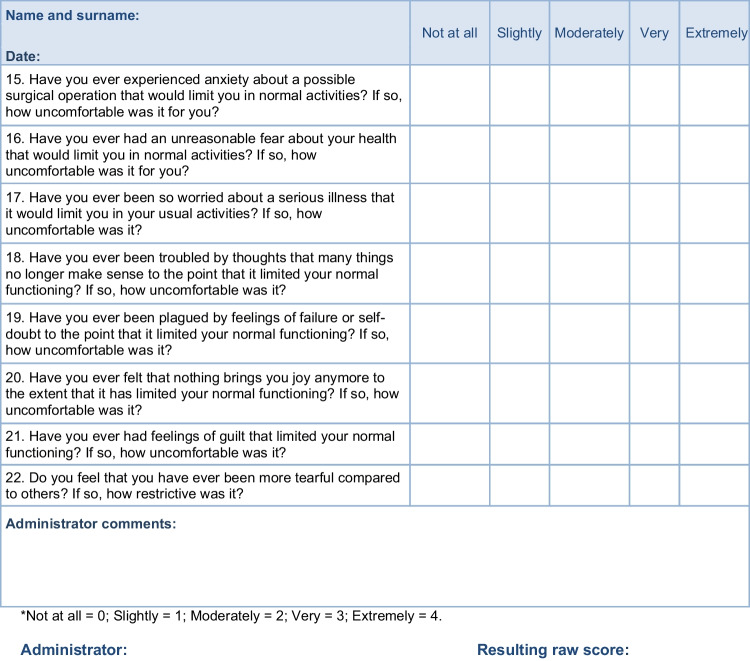
The Risk of Pain Chronification Questionnaire (RPCQ). For each question, only one option should be ticked that describes the reality the best*

**Table 2:**
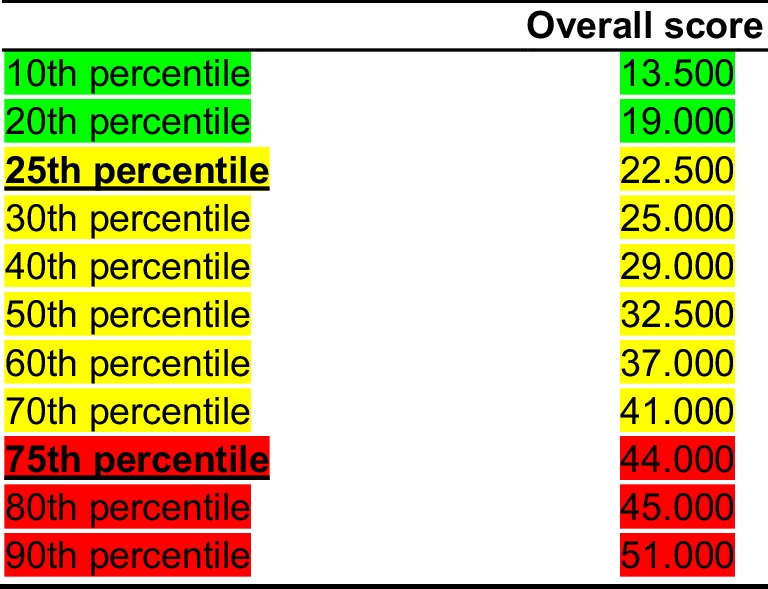
Score and references for the Slovak population. Risk of chronification and low responding to standard treatment for pain. Sum of all items scores (“Not at all” = 0; “Slightly” = 1; “Moderately” = 2; “Very” = 3; “Extremely” = 4). Risk of chronification and low responding to standard treatment for pain: Low risk—overall score ≤ 22 (green), medium risk—overall score 23–43 (yellow), and high risk—overall score ≥ 44 (red). Scores are based on percentiles for population of male and female included together. The overall score ranges from 0 to 88. The references (norms) for the Slovak population are in the table below

The health-related anxiousness and health-related depressiveness represent psychological factors in our study. The health-related anxiousness and health-related depressiveness are factors proposed by our EFA that have theoretical and empirical ground in the results of previous authors performed robust meta-analysis and systematic review to assess the role of psychological and psychosocial factors in predicting presence and intensity of chronic postsurgical pain [14]. Their results showed that depressiveness, state anxiety, trait anxiety, catastrophizing, mental health, kinesiophobia, and self-efficacy have weak but significant association with presence and intensity of chronic postsurgical pain. We are convinced that factors of health related to anxiousness and depressiveness and its items can adequately represent the typical personality traits vulnerable for experiencing depressiveness or anxiety measured by other instruments. Similarly to other studies mentioned by Giusti et al. [14], we performed linear regression which showed that the overall score of RPCQ significantly predicted the small portion of variance of severity of pain and functional ability of the patients after 6 months of standard treatment of the pain. Moreover, repeated analysis of variance showed that there was a significant difference between pre-test and post-test for reporting of the pain and functional ability according to lower or higher scores of health-related depressiveness. The respondents who scored lower on health-related depressiveness during pre-test were less likely to report severe pain or decrease in functional ability after 6 months of standard treatment (post-test). According to these results, we assume that RPCQ is a favorable candidate tool for predicting the chronification of the pain.

### Limitations

Without any doubts, our study has several limitations, and more research is needed to confirm the predictive possibilities of the RPCQ for predicting chronification of the pain in different research settings. With more respondents in groups, we could find other factors of RPCQ as significant for differences between pre-test and post-test severity of the pain and functional ability of the respondents.

## Conclusion, expert recommendations, and outlook in framework of 3PM

The novel and evidence-based risk assessment approach for pain chronification utilizing advanced survey for self-reporting patients (RPCQ) was created in the presented study. The study is aimed at the patient phenotyping and scoring of relevant risk factors followed by the patient stratification for predictive approach which allows for advancing overall pain management and targeted protection of individuals predisposed to the pain.

The proposed clusters of corresponding risk factors (items 1–22) in Table 1, described above, are highly relevant to the pain chronification and were created based on the long-term experience collected by experts at the Department of Algesiology, F.D. Roosevelt University General Hospital in Banska Bystrica, Slovakia, who elaborated it on individualized patient profiles. Overall, RPCQ scoring data demonstrate high level of validity and predictive power of the proposed survey specifically for patients persistently suffering from chronic pain after 6 months of standard anti-pain treatments applied. Noteworthy, patients with low scores recorded specifically within the fourth factor, which reflects the level of health-related depressiveness, demonstrated on one hand significantly decreased pain severity and, on the other hand, maintained their vitality after 6 months of standard anti-pain treatments. The latter is an important indicator bridging together individualized patient profile identified by RPCQ, positive therapy outcomes, and potentially maintained mitochondrial viability which might be the “game changer” in improving individual outcomes [27].

### Clinical procedure after risk identification based on a questionnaire: options for algesiological treatment

According to the achieved result (Table 2), the score of the degree of sensitization and the risk of developing chronic pain—what do we do with the given patient in clinical practice?:

1st group: low score of the degree of sensitization and risk of developing chronic pain (total score 0–22).

There is no special to be followed in terms of treatment of pain for these patients.

2nd group: moderately high score of the degree of sensitization and the risk of developing chronic pain (total score 23–43).

(2a) In the case that the patient does not suffer from any significant pain then for the gradual adjustment of a moderately high degree of CNS sensitization, it is necessary to apply to the patient the low doses of antidepressants (AD) of the group of SSRIs (selective serotonin reuptake inhibitor) or SNRIs (serotonin and norepinephrine reuptake inhibitor) (for long-term and regular use (general practitioner, physician of the PPPM clinic, and others) together with an explaining, motivational, and comprehensible instruction using the ERAS (enhanced recovery after surgery program) information leaflet. An examination by a clinical psychologist and possible psychotherapy to release the negative contents of the subconscious as the most common cause of CNS sensitization is also suitable.

(2b) In the case that the patient already suffers from chronic pain, a standard algesiology examination is required with the use of complex individually selected pain pharmacotherapy, including antidepressant group SSRIs or SNRI. An examination by a clinical psychologist and possible psychotherapy to release the negative contents of the subconscious as the most common cause of CNS sensitization is also suitable.

3rd group: high score of the degree of sensitization and risk of developing chronic pain (total score 44–88).

(3a) In the case that the patient does not suffer from any significant pain then for the gradual adjustment of a high degree of CNS sensitization, it is necessary to apply the same procedure as in the case (2a) mentioned above.

(3b) In case the patient already suffers from chronic pain, a standard algesiological examination is necessary with the deployment of complex algesiological therapy using a combination of a wide range of pharmacotherapy, non-pharmacological techniques (such as targeted rehabilitation, psychotherapy, and acupuncture, which can often intervene in the process of chronic pain treatment), and invasive pain treatment techniques. In the pharmacotherapy of chronic pain, we most often use a suitable and individually selected combination of non-opioid and opioid analgesics and adjuvant drugs, primarily antidepressant group SSRIs or SNRIs and other psychopharmaceuticals but also anticonvulsants, muscle relaxants, local anesthetics, ketamine, magnesium, B and D vitamins, and other pharmacological interventions. An examination by a clinical psychologist and possible psychotherapy to release the negative contents of the subconscious as the most common cause of CNS sensitization is also appropriate.

### The status quo in psychological stress, pain, mitochondrial stress, and proposed follow-up

In the context of chronic pain treatment, it is important to emphasize that to this end, the majority of painkillers (including aspirin) are considered a medication leading to pronounced mitochondrial stress and can cause mitochondrial burnout with severe health adverse effects [28, 29]. Therefore, the RPCQ-based discovery is crucial, clearly indicating that patient stratification is possible, distinguishing between (a) vulnerable individuals who are particularly susceptible vs. (b) individuals who are rather resistant towards mitochondrial stress under therapy conditions applied.

Therefore, in the follow-up research, an identification of specific patient phenotypes corresponding either to vulnerable individuals or to individuals resistant to mitochondrial stress is strongly recommended and we anticipate:

- Further increase in predictive power of the presented RPCQ-based approach
- Providing evidence-based indication for treatment algorithms tailored to individualized patient profiles in a holistic manner
- And improving individual outcomes by targeted rehabilitation focused on mitochondrial health in vulnerable groups

The proposed approach follows the principles of 3PM [30], meeting patient needs in a cost-effective manner and promoting the paradigm change from reactive medical services to the advanced predictive, preventive, and personalized approach in overall pain management.

In terms of in-depth patient stratification, increased stress sensitivity may be crucial for the chronic pain-associated phenotyping, since the low grade (sterile) systemic inflammation is considered characteristic for stress-sensitive individuals and, on the other hand, is attributed to the pain chronification [31]. Furthermore, psychological aspects play an important role in both low-grade inflammation and pain chronification [32]. Further, at molecular level, shifted regulation of the senses-associated receptors may be systemically linked to the level of pain perception that can be exemplified by FSP individuals demonstrating also phenotype-specific psycho-social and behavioral patterns [6–9]. Contextually, a multi-professional protection of vulnerable individuals against the pain chronification is strongly recommended for the protection of vulnerable individuals against the pain chronification.

## Supplementary Information

Below is the link to the electronic supplementary material.

Supplementary file1 (DOC 152 kb)

## Data Availability

No datasets were generated or analysed during the current study.

## Abbreviations

3PM/PPPM: Predictive, preventive, and personalized medicine
AD: Antidepressant
ALBPSQ: Acute Low Back Pain Screening Questionnaire
BPI-SF: The Brief Pain Inventory-Short Form
CNS: Central nervous system
df: Degrees of freedom
DM: Diabetes mellitus
EFA: Exploratory factor analysis
EPMA: European Association for Predictive, Preventive, and Personalized Medicine
ERAS: Enhanced recovery after surgery
FSP: Flammer syndrome phenotype
GIT: Gastro-intestinal tract
HIV/AIDS: Human immunodeficiency virus/acquired immunodeficiency syndrome
JASP: Statistical software at https://jasp-stats.org/
KMO: Kaiser-Meyer-Olkin
OMPG: Örebro Musculoskeletal Pain Questionnaire
p: *p*-Value
*R*^2^: Coefficient of determination
RPCQ: Risk of Pain Chronification Questionnaire
SNRI: Serotonin and norepinephrine reuptake inhibitor
SSRI: Selective serotonin reuptake inhibitor
TOL: Tolerance
VIF: Variance inflation factor
*χ*2: Chi-square statistics
α: Internal consistency statistics
