# Supplementary material for: Pain chronification risk assessment: advanced phenotyping and scoring for prediction and treatments tailored to individualized patient profile
Source: EPMA J. 2024 Nov 15;15(4):739–50. doi: 10.1007/s13167-024-00383-3 (PMC11612039; doi:10.1007/s13167-024-00383-3)
Supplement: Supplementary file 1 — Supplementary file1 (DOC 152 kb) [file 13167_2024_383_MOESM1_ESM.doc]

**Supplementary Table 1. Principal axis factoring for a four-factor solution using oblique rotation ordered by decreasing correlation values**

| **Items (questions)** | **Health related depresiveness** | **Health related anxiousness** | **Severe pain and sensitization** | **Visceral somatization** |
| --- | --- | --- | --- | --- |
| 20. Have you ever felt that nothing brings you joy anymore to the extent that it has limited your normal functioning? If so, how uncomfortable was it? | 0.864 |  |  |  |
| 21. Have you ever had feelings of guilt that limited your normal functioning? If so, how uncomfortable was it? | 0.808 |  |  |  |
| 19. Have you ever been plagued by feelings of failure or self-doubt to the point that it limited your normal functioning? If so, how uncomfortable was it? | 0.750 |  |  |  |
| 18. Have you ever been troubled by thoughts that many things no longer make sense to the point that it limited your normal functioning? If so, how uncomfortable was it? | 0.731 |  |  |  |
| 22. Do you feel that you have ever been more tearful compared to others? If so, how restrictive was it? | 0.685 |  |  |  |
| 14. Have you ever had disturbing thoughts about possible hospitalization that would limit you in normal activities? If so, how uncomfortable was it for you? |  | 0.862 |  |  |
| 15. Have you ever experienced anxiety about a possible surgical operation that would limit you in normal activities? If so, how uncomfortable was it for you? |  | 0.786 |  |  |
| 13. Have you ever had such anxiety that you could not concentrate on daily activities or work? If so, how uncomfortable was it for you? |  | 0.689 |  |  |
| 16. Have you ever had an unreasonable fear about your health that would limit you in normal activities? If so, how uncomfortable was it for you? |  | 0.675 |  |  |
| 17. Have you ever been so worried about a serious illness that it would limit you in your usual activities? If so, how uncomfortable was it? |  | 0.460 |  |  |
| 12. Have you ever visited several doctors on your own initiative with the same problem despite repeated negative examination results? If so, how uncomfortable was it for you? |  | 0.403 |  |  |
| 2. Have you ever experienced a sudden and extremely intense physical pain (e.g. during an accident) that significantly worsened your perception of pain? If so, how much has your perception of the new pain worsened? |  |  | 0.549 |  |
| 5. Have you ever been bothered by excessive muscle tension in areas other than the sacral and neck muscles? If so, how uncomfortable was it for you? |  |  | 0.536 |  |
| 10. Have you ever experienced excessive sensitivity to weather changes (e.g. headaches, pressure changes, dizziness, joint pain, etc.)? If so, how uncomfortable was it for you? |  |  | 0.515 |  |
| 1. Have you ever experienced any severe pain? If so, how uncomfortable was it for you? |  |  | 0.506 |  |
| 3. Have you ever been bothered by excessive muscle tension in the cervical spine (neck) area? If so, how uncomfortable was it for you? |  |  | 0.495 |  |
| 11. Has it happened to you that after pain treatment (e.g. drugs, rehabilitation, surgery) there was no expected improvement? If so, how uncomfortable was it for you? |  |  | 0.466 |  |
| 4. Have you ever been bothered by excessive muscle tension in the lower back area? If so, how uncomfortable was it for you? |  |  | 0.447 |  |
| 6. Have you ever had trouble urinating excessively? If so, how uncomfortable was it for you? |  |  |  | 0.679 |
| 7. Have you ever had difficulty with pain while urinating? If so, how uncomfortable was it for you? |  |  |  | 0.637 |
| 9. Have you ever had difficulty with bowel movements (diarrhea, constipation)? If so, how uncomfortable was it for you? |  |  |  | 0.457 |
| 8. Have you ever had problems with pain in the genital area? If so, how uncomfortable was it for you? |  |  |  | 0.401 |

**Supplementary Table 2. Correlation coefficients among the four obtained factors: Health-related depresivness; Healt-related anxiousness; Sever pain and sensitisation; Visceral somatization of RPCQ**

|  | | | | | | | | | | |
| --- | --- | --- | --- | --- | --- | --- | --- | --- | --- | --- |
|  | | **Health-related depressiveness** | | | **Healt- related anxiousness** | | **Severe pain and sensitization** | | **Visceral somatization** | |
| Health-related depressiveness |  | |  |  | 0.686 |  | 0.341 |  | 0.282 |  |
| Health -related anxiousness |  | | 0.686 |  |  |  | 0.384 |  | 0.289 |  |
| Severe pain and sensitization |  | | 0.341 |  | 0.384 |  |  |  | 0.288 |  |
| Visceral somatization |  | | 0.282 |  | 0.289 |  | 0.288 |  |  |  |
|  | | | | | | | | | | |

**Supplementary Table 3. Descriptive statistics of the variables included into multiple regression analysis for predicting severity of the pain and functional ability after 6 months of standard treatment**

| **Variables** | **Number of participants** | **Mean score** | **Standard Deviation** |
| --- | --- | --- | --- |
| Severity of the pain after 6 months | 116 | 5.274 | 2.301 |
| Functional ability | 116 | 4.388 | 2.257 |
| Age | 116 | 59.319 | 14.603 |
| Severe pain and sensitization | 116 | 2.251 | 0.718 |
| Visceral somatization | 116 | 0.866 | 0.749 |
| The health related anxiousness | 116 | 1.415 | 0.918 |
| The health related depressiveness | 116 | 1.078 | 0.972 |

**Supplementary Table 4. Multiple linear regression predicting severity of the pain after 6 months of standard treatment**

| **Model** | **Variables** | **Unstandardized**  **value** | **Standardized Error** | **Standardized**  **value** | **t** | **p-value** |
| --- | --- | --- | --- | --- | --- | --- |
| H_0_ | (Intercept) | 5.274 | 0.214 |  | 24.685 | < 0.001 |
| H_1_ | (Intercept) | 1.587 | 1.053 |  | 1.507 | 0.135 |
|  | Age | 0.027 | 0.014 | 0.172 | 1.970 | 0.051 |
|  | Severe pain and sensitization | 0.528 | 0.325 | 0.165 | 1.623 | 0.107 |
|  | Visceral somatization | 0.245 | 0.300 | 0.080 | 0.816 | 0.416 |
|  | Health related anxiousness | 0.053 | 0.326 | 0.021 | 0.162 | 0.872 |
|  | Health related depressiveness | 0.561 | 0.302 | 0.237 | 1.857 | 0.066 |
| t: t-test statistic, p-value: probability rate | | | | | | |

**Supplementary Table 5. Multiple linear regression predicting functional ability after 6 months standard treatment**

| **Model** | **Variables** | **Unstandardized**  **value** | **Standardized Error** | **Standardized**  **value** | **t** | **p-value** |
| --- | --- | --- | --- | --- | --- | --- |
| H_0_ | (Intercept) | 4.388 | 0.210 |  | 20.943 | <0 .001 |
| H_1_ | (Intercept) | 2.450 | 1.075 |  | 2.279 | 0.025 |
|  | Age | 0.002 | 0.014 | 0.011 | 0.116 | 0.908 |
|  | Severe pain and sensitization | 0.441 | 0.332 | 0.140 | 1.329 | 0.187 |
|  | Visceral somatization | 0.361 | 0.307 | 0.120 | 1.175 | 0.242 |
|  | Health related anxiousness | 0.046 | 0.333 | 0.019 | 0.138 | 0.890 |
|  | Health related depressiveness | 0.436 | 0.309 | 0.188 | 1.415 | 0.160 |
| Note. t: t-test statistic, p-value: probability rate | | | | | | |

**Supplementary Table 6. T-test result comparing scores of severity of the pain and functional ability vs. same scores after 6 months of standard treatment**

| **Variables** | **Start of the standard treatment** | | | **After six months of standard treatment** | | | **t-test** |
| --- | --- | --- | --- | --- | --- | --- | --- |
|  | **M** | **SD** | **n** | **M** | **SD** | **n** |  |
| Severity of the pain | 6.143 | 1.967 | 116 | 5.274 | 2.301 | 116 | 3.798** |
| Functional ability | 5.382 | 2.281 | 116 | 4.388 | 2.257 | 116 | 3.850** |
| Note: M: Mean; SD: Standard deviation; n: Sample size, **p<0.01 | | | | | | | |

**Supplementary Table 7. Repeated measures ANOVA: Severity of the pain at the start of the standard treatment and after according to low or high score on health related depressiveness**

| **Variables (N)** | **Sum of Squares** | **df** | **Mean Square** | **F-test** | **p** |
| --- | --- | --- | --- | --- | --- |
| Severity of the pain | 71.128 | 1 | 71.128 | 35.990 | < .001 |
| Severity of the pain ✻ Health related depressiveness | 9.616 | 1 | 9.616 | 4.866 | 0.031 |
| Residuals | 122.531 | 62 | 1.976 |  |  |
|  | | | | | |
| Note*.* p-value: probability rate, df: degrees of freedom | | | | | |

**Supplementary Table 8.. Post hoc comparisons between pretests and posttests for severity of the pain according to low or high score on RCPQ**

|  | | **Mean Difference** | **SE** | **t** | **pholm** |
| --- | --- | --- | --- | --- | --- |
| Pretest - Severity of the pain (Low score of RCPQ) | Pretest - Severity of the pain (High score of RCPQ) | -1.321 | 0.531 | -2.489 | 0.043 |
|  | Posttest - Severity of the pain (Low score of RCPQ) | 1.552 | 0.421 | 3.686 | 0.003 |
|  | Posttest - Severity of the pain (High score of RCPQ) | -0.282 | 0.531 | -0.532 | 0.596 |
| Pretest - Severity of the pain (High score of RCPQ) | Posttest - Severity of the pain (Low score of RCPQ) | 2.873 | 0.531 | 5.411 | < .001 |
|  | Posttest - Severity of the pain (High score of RCPQ) | 1.039 | 0.414 | 2.510 | 0.043 |
| Posttest - Severity of the pain (Low score of RCPQ) | Posttest - Severity of the pain (High score of RCPQ) | -1.834 | 0.531 | -3.455 | 0.003 |
|  | | | | | |
| Note. SE: standard error; t: t-test statistic, pholm- p-value according to Holm method | | | | | |

**Supplementary Table 9. Post hoc comparisons between pretests and posttests for functional ability according to low or high score on RCPQ**

|  | | **Mean Difference** | **SE** | **t** | **pholm** |
| --- | --- | --- | --- | --- | --- |
| Pretest - Functional ability (Low score of RCPQ) | Pretest - Functional ability (High score of RCPQ) | -2.558 | 0.569 | -4.499 | < .001 |
|  | Posttest - Functional ability (Low score of RCPQ) | 1.299 | 0.487 | 2.669 | 0.020 |
|  | Posttest Functional ability (High score of RCPQ) | -0.806 | 0.569 | -1.417 | 0.159 |
| Pretest - Severity of the pain (High score of RCPQ) | Posttest - Functional ability (Low score of RCPQ) | 3.857 | 0.569 | 6.782 | < .001 |
|  | Posttest - Functional ability (High score of RCPQ) | 1.752 | 0.478 | 3.663 | 0.002 |
| Posttest - Functional ability (Low score of RCPQ) | Posttest - Functional ability (High score of RCPQ) | -2.105 | 0.569 | -3.701 | 0.001 |
| Note. SE: standard error; t: t-test statistic, H_0_: the null hypothesis, H₁: alternative hypothesis, pholm- p-value according to Holm method | | | | | |
|  | | | | | |
|  | | | | | |

**Supplementary Table 10. Post hoc comparisons between pre-tests and post-tests for severity of the pain according to low or high score on health related depressiveness**

|  |  | **Mean Difference** | **SE** | **t** | **Cohen's d** | **pholm** |
| --- | --- | --- | --- | --- | --- | --- |
| Pretest - Severity of the pain (Low score of health related depresiveness) | Pretest - Severity of the pain  (High score of health related depresiveness) | -1.060 | 0.516 | -2.053 | -0.513 | 0.086 |
|  | Post test - Severity of the pain  (Low score of health related depressiveness) | 2.039 | 0.351 | 5.802 | 0.987 | < .001 |
|  | Post test - Severity of the pain  (High score of health related depressiveness) | -0.117 | 0.516 | -0.227 | -0.057 | 0.821 |
| Pretest - Severity of the pain  (High score of health related depressiveness) | Post test - Severity of the pain  (Low score of health related depressiveness) | 3.099 | 0.516 | 6.002 | 1.501 | < .001 |
|  | Post test  (High score of health related depressiveness) | 0.943 | 0.351 | 2.682 | 0.456 | 0.028 |
| Post test - Severity of the pain  (Low score of health related depressiveness) | Post test - Severity of the pain  (High score of health related depressiveness) | -2.156 | 0.516 | -4.176 | -1.044 | < .001 |
|  | | | | | | |
| Note. SE: standard error; t: t-test statistic, H_0_: the null hypothesis, H₁: alternative hypothesis, Cohen’s d- standardized mean difference, pholm- p-value according to Holm method | | | | | | |
